# The causal effects of inflammatory and autoimmune skin diseases on thyroid diseases: evidence from Mendelian randomization study

**DOI:** 10.3389/fendo.2024.1388047

**Published:** 2024-09-02

**Authors:** Ruixuan You, Jiayue Duan, Yong Zhou, Jiangfan Yu, Puyu Zou, Yi Wei, Ke Chai, Zhuotong Zeng, Yangfan Xiao, Lingqing Yuan, Rong Xiao

**Affiliations:** ^1^ Department of Dermatology, The Second Xiangya Hospital of Central South University, Changsha, China; ^2^ Hunan Key Laboratory of Medical Epigenetics, The Second Xiangya Hospital of Central South University, Changsha, China; ^3^ Clinical Medical Research Center for Systemic Autoimmune Diseases in Hunan Province, The Second Xiangya Hospital of Central South University, Changsha, China; ^4^ Department of Endocrinology, Key Laboratory of Endocrinology, Ministry of Health, Peking Union Medical College Hospital, Chinese Academy of Medical Sciences & Peking Union Medical College, Beijing, China; ^5^ Department of Cardiovascular Medicine, The Second Xiangya Hospital of Central South University, Changsha, China; ^6^ Clinical Nursing Teaching and Research Section, The Second Xiangya Hospital of Central South University, Changsha, China; ^7^ Department of Anesthesiology, The Second Xiangya Hospital of Central South University, Changsha, China; ^8^ National Clinical Research Center for Metabolic Disease, Hunan Provincial Key Laboratory of Metabolic Bone Diseases, The Second Xiangya Hospital of Central South University, Changsha, China; ^9^ Department of Endocrinology and Metabolism, The Second Xiangya Hospital of Central South University, Changsha, China

**Keywords:** inflammatory skin diseases, autoimmune skin diseases, hypothyroidism, hyperthyroidism, Mendelian randomization

## Abstract

**Background:**

To clarify the controversy between inflammatory or autoimmune skin diseases and thyroid diseases, we performed two-sample Mendelian randomization (MR) analyses.

**Participants:**

Genetic data on factors associated with atopic dermatitis (AD, n=40,835), seborrheic dermatitis (SD, n=339,277), acne (n=363,927), rosacea (n=299,421), urticaria (n=374,758), psoriasis (n=373,338), psoriasis vulgaris (n=369,830), systemic lupus erythematosus (SLE, n=14,267), vitiligo (n=353,348), alopecia areata (AA, n=361,822), pemphigus (n=375,929), bullous pemphigoid (BP, n=376,274), systemic sclerosis (SSc, n=376,864), localized scleroderma (LS, n=353,449), hypothyroidism (n=314,995 or n=337,159), and hyperthyroidism (n=281,683 or n=337,159) were derived from genome-wide association summary statistics of European ancestry.

**Main measures:**

The inverse variance weighted method was employed to obtain the causal estimates of inflammatory or autoimmune skin diseases on the risk of thyroid diseases, complemented by MR-Egger, weighted median, and MR-pleiotropy residual sum and outlier (MR-PRESSO).

**Key results:**

AD, SLE, SD, and psoriasis vulgaris were associated with an increased risk of hypothyroidism, whereas BP was associated with a lower risk of hypothyroidism (all with p < 0.05). The multivariable MR analyses showed that AD (OR = 1.053; 95%CI: 1.015-1.092; p = 0.006), SLE (OR = 1.093; 95%CI: 1.059-1.127; p < 0.001), and SD (OR = 1.006; 95%CI: 1.002-1.010; p = 0.006) independently and predominately contributed to the genetic causal effect on hypothyroidism after adjusting for smoking. The results showed no causal effects of inflammatory or autoimmune skin diseases on hyperthyroidism.

**Conclusion:**

The findings showed a causal effect of AD, SLE, SD on hypothyroidism, but further investigations should be conducted to explore the pathogenic mechanisms underlying these relationships.

## Introduction

The skin is located at the boundary between the body and the external environment and is the first line of defense against pathogen invasion (1). Local keratinocytes and skin-resident immune cells initiate innate immune mechanisms when the epidermal barrier is disrupted by intrinsic or extrinsic factors, to clear the infection, promote wound healing, and activate adaptive immunity (2). However, once the skin immune homeostasis is dysregulated, excessive release of cytokines and chemokines forms an inflammatory cascade, causing uncontrolled activation of T cells, the humoral immune system or innate immunity, resulting in inflammatory or autoimmune skin diseases (3).

Autoimmune thyroid disease (AITD) manifests as an organ-specific autoimmune disorder triggered by a T cell attack on the thyroid due to an imbalance in immune system homeostasis. The invasion of lymphocytes into the thyroid, mainly infiltration of activated T cells, leads to gradual replacement of thyroid tissue, which impairs thyroid hormone synthesis (4). In addition, recruited helper T lymphocytes 1 (Th1) produce IFN-γ and TNF-α and then thyroid cells respond and secrete CXCL10, promoting infiltration of more lymphocytes, thereby creating a positive feedback loop that triggers and amplifies the autoimmune process (5). In the early stage of AITD, the prevalent immune response is Th1 immune response, which changes to Th2 immune response in the late stage (6). The prevailing clinical hallmarks of AITD are hyperthyroidism and hypothyroidism (7, 8). In addition, toxic compounds in tobacco smoke, such as nicotine, thiocyanate and benzopyrene, can exert pro- and anti-thyroid effects by affecting iodine uptake and organification or modulating the sympathetic nervous system activity (9), which subsequently cause AITD.

Previous epidemiological findings revealed that AITD was reported to have a higher prevalence among inflammatory (dermatitis, urticaria, etc.) or autoimmune skin diseases ((systemic lupus erythematosus (SLE), vitiligo, pemphigus, systemic sclerosis (SSc), etc.) patients compared to the general population (10), indicating that common underlying pathophysiological mechanisms may exist in the skin-thyroid axis. However, due to the limitations of observational studies, only the high comorbidity rate of these two diseases can be concluded, and a causal effect of inflammatory or autoimmune skin diseases on AITD has not been fully elucidated.

Mendelian Randomization (MR) represents a novel epidemiological research design that employs genetic variants (single-nucleotide polymorphisms (SNPs)) as instrumental variables (IVs) to infer the causal relationship between the outcome and exposure (11). The use of MR can minimize the interference of unmeasured confounding factors and reverse causation bias owing to the random distribution of genetic variants at conception. MR analysis can save considerable human resources, time and costs compared with conventional observational studies and randomized controlled trials (RCTs). Multivariate MR (MVMR) serves an extension of MR designed to evaluate the causal effects of two or more exposures on the outcome (12).

Therefore, the aim of this study was to estimate the causal association between inflammatory or autoimmune skin diseases and thyroid dysfunction using a two-sample MR framework to provide theoretical support for clinical decision-making. We also performed MVMR to evaluate the mediating effect of smoking on the relationship between inflammatory or autoimmune skin diseases and thyroid dysfunction.

## Methods

### MR design

**Figure 1:**
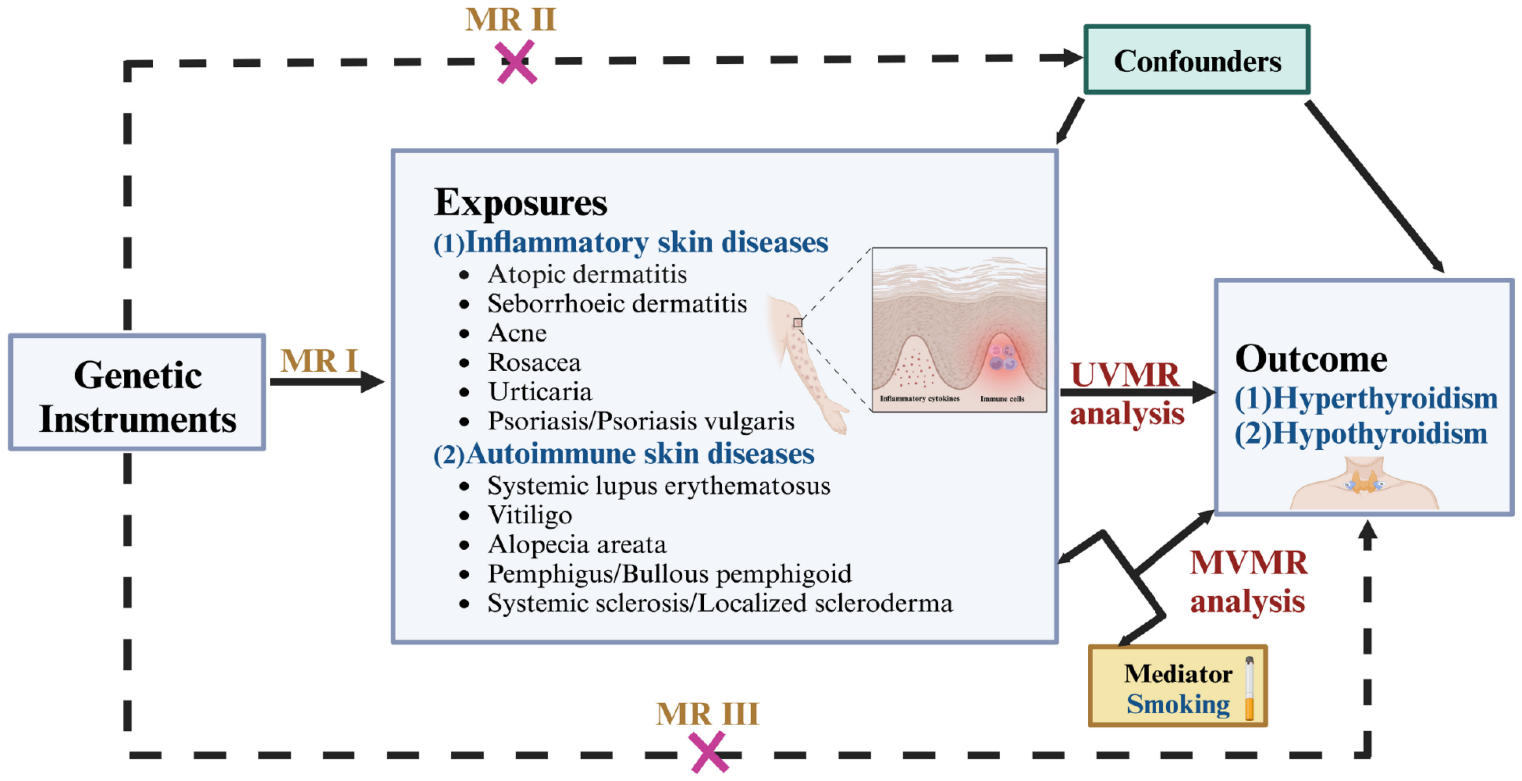
Overview of the design and procedures used in this MR study. There are three key assumptions of MR design. MR assumptions I: IVs are highly associated with the exposure; MR assumptions II: There is no correlation between IVs and confounders; MR assumptions III: IVs affect the outcome only through the exposure. MR, Mendelian randomization; UVMR, univariable MR; MVMR, multivariate MR.

Based on publicly available genome-wide association summary (GWAS) data from European descent, we performed a two sample MR analysis to assess the causal association between inflammatory or autoimmune skin diseases and thyroid dysfunction. Ethical approval was not necessary for this study because the datasets used were derived from publicly published summary-level GWAS data that had received the relevant ethical approval. A summary of the design and procedures used in this study is presented in **Figure 1**. In MR analysis, IVs must satisfy three essential criteria to obtain reliable inferences: I) Relevance: IVs are highly associated with exposure; II) Independence: There is no correlation between IVs and confounders; III) Exclusion restriction: IVs affect the outcome only through the exposure (13). The findings in this study are reported following the STROBE-MR guidelines.

### Data sources

The skin diseases were grouped into two categories: (i) inflammatory skin diseases, including atopic dermatitis (AD), seborrheic dermatitis (SD), acne, rosacea, urticaria, psoriasis and psoriasis vulgaris; (ii) autoimmune skin diseases, including SLE, vitiligo, alopecia areata (AA), pemphigus, bullous pemphigoid (BP), SSc, and localized scleroderma (LS). We extracted IVs of traits from five sources: (i) EArly Genetics and Lifecourse Epidemiology (EAGLE) Consortium (https://www.eagle-consortium.org/); (ii) Medical Research Council Integrative Epidemiology Unit (MRC-IEU) (https://gwas.mrcieu.ac.uk/); (iii) Neale Lab (http://www.nealelab.is/uk-biobank); (iv) FinnGen Biobank (https://www.finngen.fi/en) (14); (v) GWAS and Sequencing Consortium of Alcohol and Nicotine Use (GSCAN) (https://conservancy.umn.edu/handle/11299/201564). Details on the data sources used in this study are presented in **Table 1**.

**Table 1:** GWAS datasets in the Mendelian randomization study.

Phenotype	Consortium	Ancestry	Sample size (case/control)	Year of publication	PMID
Exposure					
Inflammatory skin diseases					
Atopic dermatitis	EAGLE	European	10,788/30,047	2014	26482879
Seborrheic dermatitis	FinnGen	European	2,688/336,589	2023	36653562
Acne	FinnGen	European	2,787/361,140	2023	36653562
Rosacea	FinnGen	European	1,877/297,544	2022	NA
Urticaria	FinnGen	European	1,0175/364,583	2023	36653562
Psoriasis	FinnGen	European	9,267/364,071	2023	36653562
Psoriasis vulgaris	FinnGen	European	5,759/364,071	2023	36653562
Autoimmune skin diseases					
Systemic lupus erythematosus	MRC-IEU	European	5,201/9,066	2015	26502338
Vitiligo	FinnGen	European	260/353,088	2023	36653562
Alopecia areata	FinnGen	European	682/361,140	2023	36653562
Pemphigus	FinnGen	European	162/375,767	2023	36653562
Bullous pemphigoid	FinnGen	European	507/375,767	2023	36653562
Systemic sclerosis	FinnGen	European	194/376,670	2023	36653562
Localized scleroderma	FinnGen	European	361/353,088	2023	36653562
Outcome					
Phenotype of hypothyroidism					
Hypothyroidism, strict autoimmune	FinnGen	European	40,926/274,069	2023	36653562
Hypothyroidism/myxoedema	Neale Lab	European	16,376/320,783	2017	NA
Phenotype of hyperthyroidism					
Autoimmune hyperthyroidism	FinnGen	European	1,828/279,855	2023	36653562
Hyperthyroidism/thyrotoxicosis	Neale Lab	European	2,547/334,612	2017	NA
Mediator					
Phenotype of smoking					
Smoking initiation	GSCAN	European	311,629/321,173	2019	30643251
Smoking dependency	FinnGen	European	2,175/373,906	2023	36653562

PMID, Pubmed Unique Identifier; NA, not available; MRC-IEU, Medical Research Council Integrative Epidemiology Unit; GSCAN, GWAS and Sequencing Consortium of Alcohol and Nicotine Use.

### Selection of genetic variants

**Figure 2:**
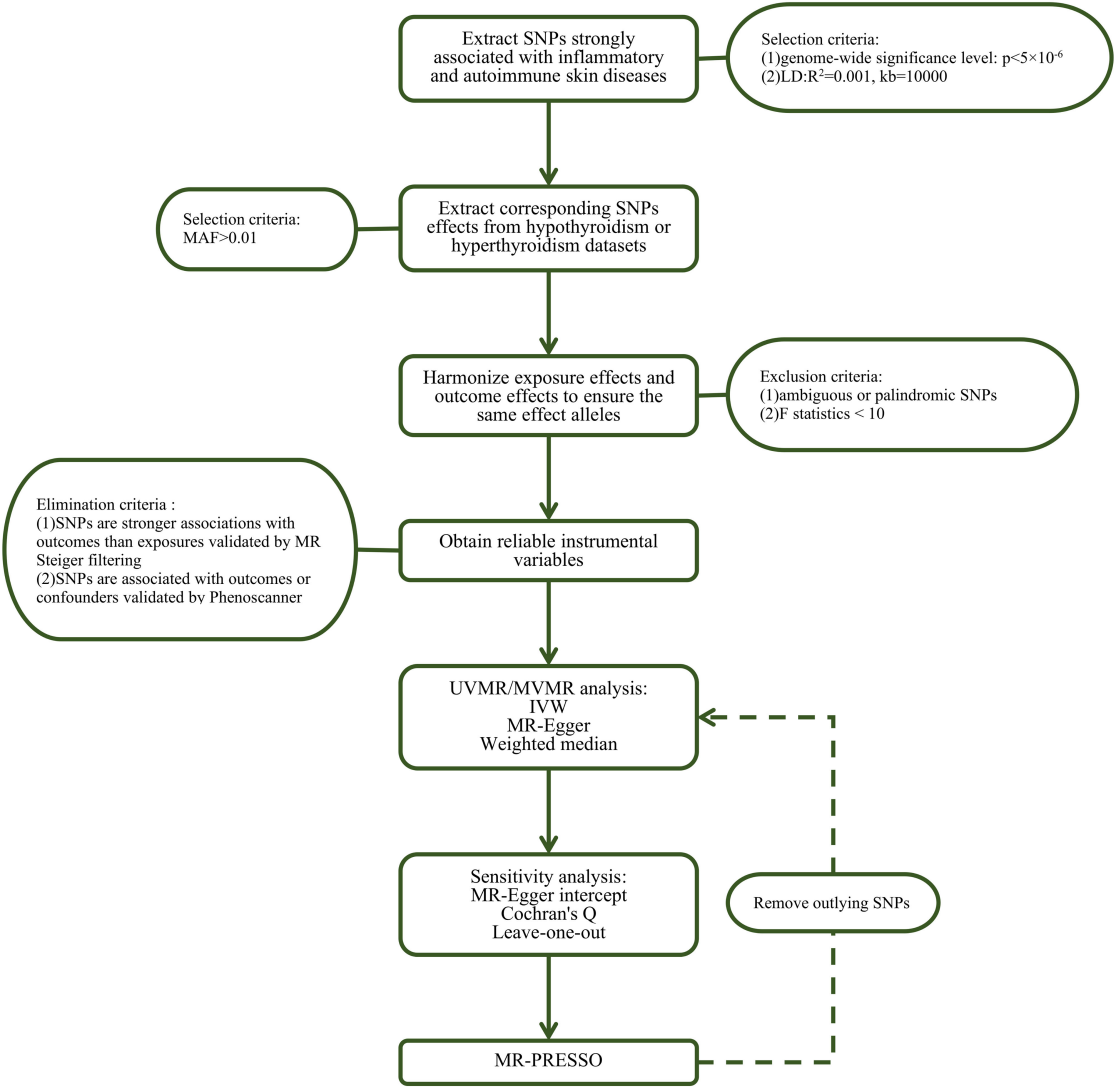
Flow chart about the analytical methods and how the MR analysis was performed step-by-step. SNP, single nucleotide polymorphism; MAF, minor allele frequency; MR, Mendelian randomization; UVMR, univariable MR; MVMR, multivariate MR; IVW, inverse variance weighted; MR-PRESSO, Mendelian randomization pleiotropy residual sum and outlier.

We performed a series of quality control steps to select instrumental SNPs, as shown in **Figure 2**. Firstly, we adjusted the significance level and extracted SNPs associated with inflammatory or autoimmune skin diseases with genome-wide significance (P < 5×10^−6^) to ensure high statistical power of the study. Secondly, we performed clumping with R^2^<0.001 and a window size of 10,000 kb to exclude SNPs linked to linkage disequilibrium (LD). Thirdly, we extracted data for the selected SNPs from the outcome thyroid dysfunction GWAS summary statistics with a minor allele frequency > 0.01. Subsequently, SNPs with F-statistics < 10 or the palindromic and ambiguous SNPs were excluded during harmonization of exposure and outcome effects. Moreover, SNPs with secondary phenotypes associated with the outcome or confounding factors, as shown in the Phenoscanner database (http://www.phenoscanner.medschl.cam.ac.uk/), including but not limited to iodine nutrition, ethnicity, autoimmune disorders, stress, selenium and vitamin D deficiency (15, 16), were excluded to eliminate pleiotropic effects. Finally, MR Steiger filtering was conducted to evaluate the direction of causality for each SNP on exposure and outcome, ensuring that the selected SNPs explained more exposure variance compared to outcome. Details on SNPs used as valid instrumental variables for the effect of inflammatory or autoimmune skin diseases on hypothyroidism or hyperthyroidism are presented in **Supplementary Tables S1**-**S28**.

We calculated the F statistic (F = beta^2^_exposure_/se^2^_exposure_) to determine presence of weak IVs. F statistic of IVs significantly greater than 10 indicated that the association between the IVs and the exposure was sufficient to minimize weak instrumental variable bias (17).

### Statistical analysis

We initially conducted two-sample univariable MR (UVMR) analyses to assess the causal effect of each exposure (inflammatory or autoimmune skin diseases) on thyroid dysfunction using the inverse variance weighted (IVW) supplemented by weighted median and MR-Egger methods. Consistency in the direction of estimates across all MR methods enhanced the reliability of the causal relationships. The fixed-effects IVW was used if there was no heterogeneity among IVs whereas the multiplicative-random-effects IVW was used when heterogeneity was detected among IVs. We also performed MVMR analyses to explore the effect of multiple exposures on the outcome after adjusting for smoking (18).

The heterogeneity among SNPs was assessed using Cochran’s Q test and funnel plots were generated for visualization, with p < 0.05 indicating potential heterogeneity. The degree of deviation of the intercept from zero for the MR-Egger regression was used as an index to evaluate the horizontal pleiotropy, with p < 0.05 indicating horizontal pleiotropy (19). The MR Pleiotropy RESidual Sum and Outlier (MR-PRESSO) test was used to evaluate the outlier SNPs with potential horizontal pleiotropy with the number of distributions set to 10,000 (20). We excluded the outliers and re-evaluated the MR effect. We eliminated the SNPs one at a time using the leave-one-out method and MR analysis performed on the remaining SNPs to determine whether each SNP could significantly influence the results to evaluate the robustness.

We visualized the MR results by generating scatter plots and conducting the leave-one-out sensitivity test.

All statistical analyses in this study were conducted in R software (version 4.3.1; R Foundation for Statistical Computing, Vienna, Austria) using TwoSampleMR (version 0.5.7), MVMR (version 0.4), and MRPRESSO (version 1.0) packages. We applied the Bonferroni correction at a statistical significance level of p < 1.79×10^−3^ (0.05/14 exposures/2 outcomes) to overcome the multiple comparison problem. P values between 1.79×10^−3^ and 0.05 indicated suggestive associations. All the MR estimates were expressed as OR values and the corresponding 95% confidence intervals (CI).

## Results

### Causal effects of inflammatory and autoimmune skin diseases on hypothyroidism

**Figure 3:**
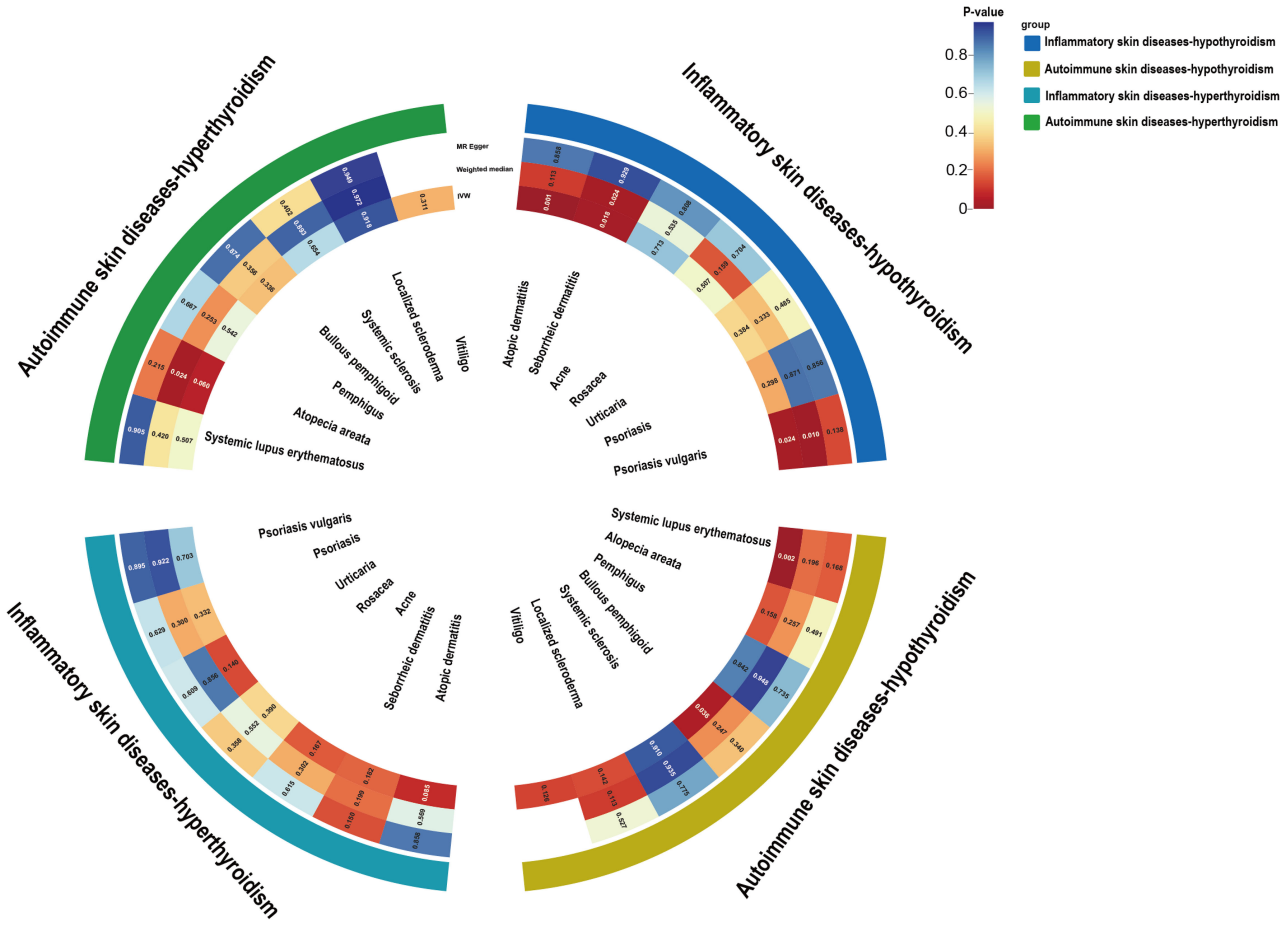
Circle heat map of the UVMR estimates for the causal associations of inflammatory or autoimmune skin diseases with hypothyroidism or hyperthyroidism. UVMR, univariable mendelian randomization; IVW, inverse variance weighted.

**Figure 4:**
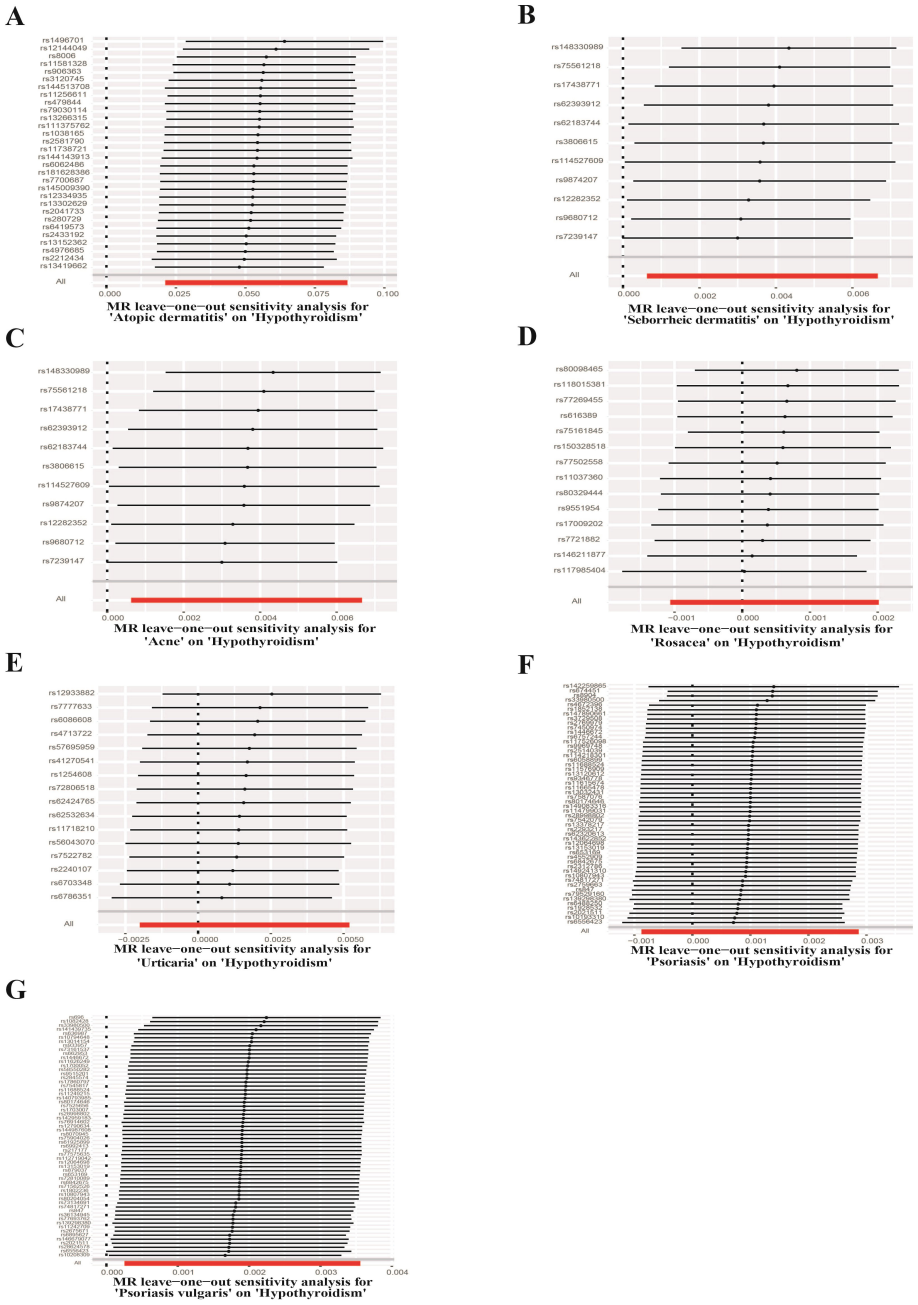
Leave-one-out test plots of causal effect estimates for inflammatory skin diseases on hypothyroidism. **(A)** Atopic dermatitis on hypothyroidism **(B)** Seborrheic dermatitis on hypothyroidism **(C)** Acne on hypothyroidism **(D)** Rosacea on hypothyroidism **(E)** Urticaria on hypothyroidism **(F)** Psoriasis on hypothyroidism **(G)** Psoriasis vulgaris on hypothyroidism.

**Figure 5:**
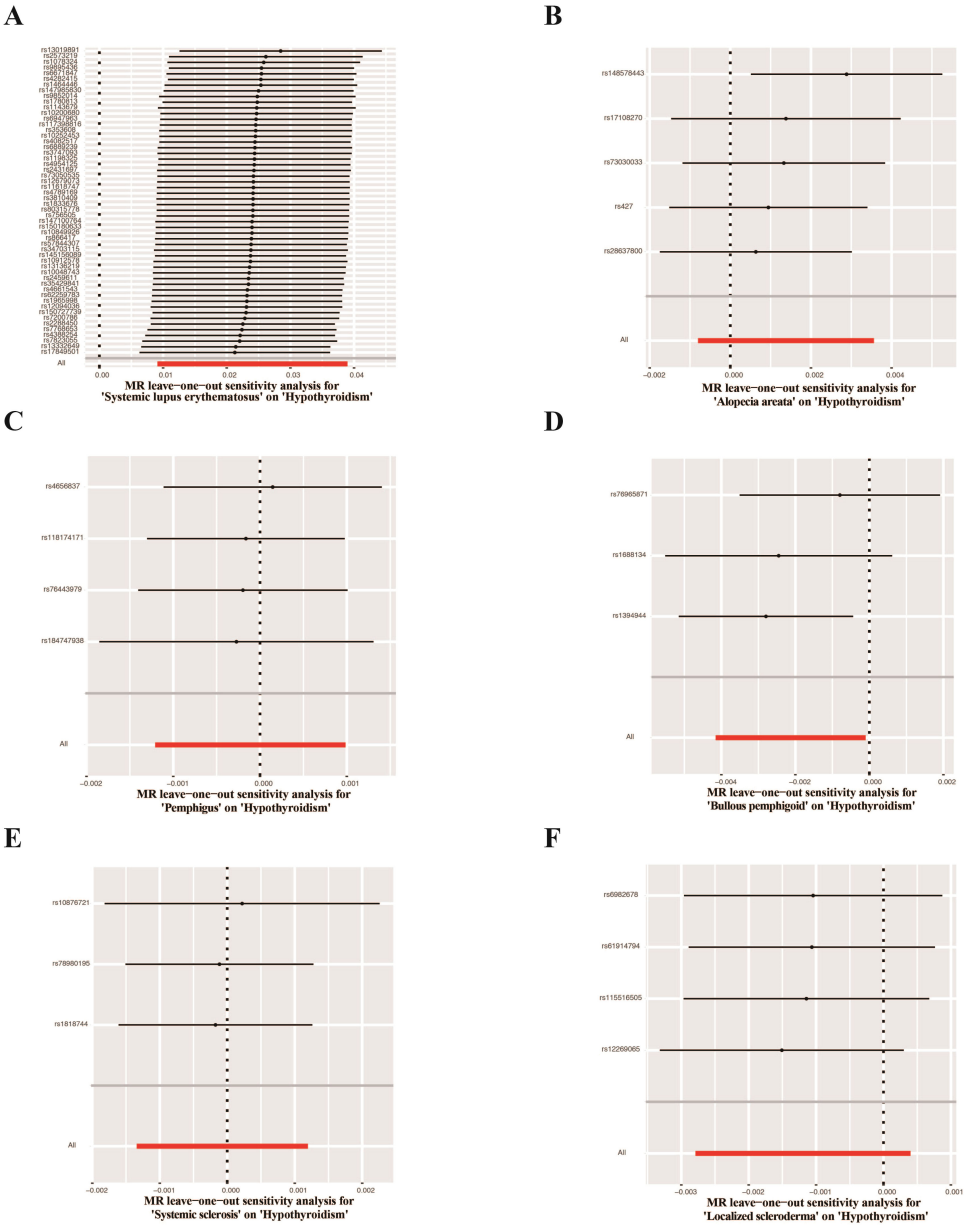
Leave-one-out test plots of causal effect estimates for autoimmune skin diseases on hypothyroidism. **(A)** Systemic lupus erythematosus on hypothyroidism **(B)** Alopecia areata on hypothyroidism **(C)** Pemphigus on hypothyroidism **(D)** Bullous pemphigoid on hypothyroidism **(E)** Systemic sclerosis on hypothyroidism **(F)** Localized scleroderma on hypothyroidism.

**Figure 6:**
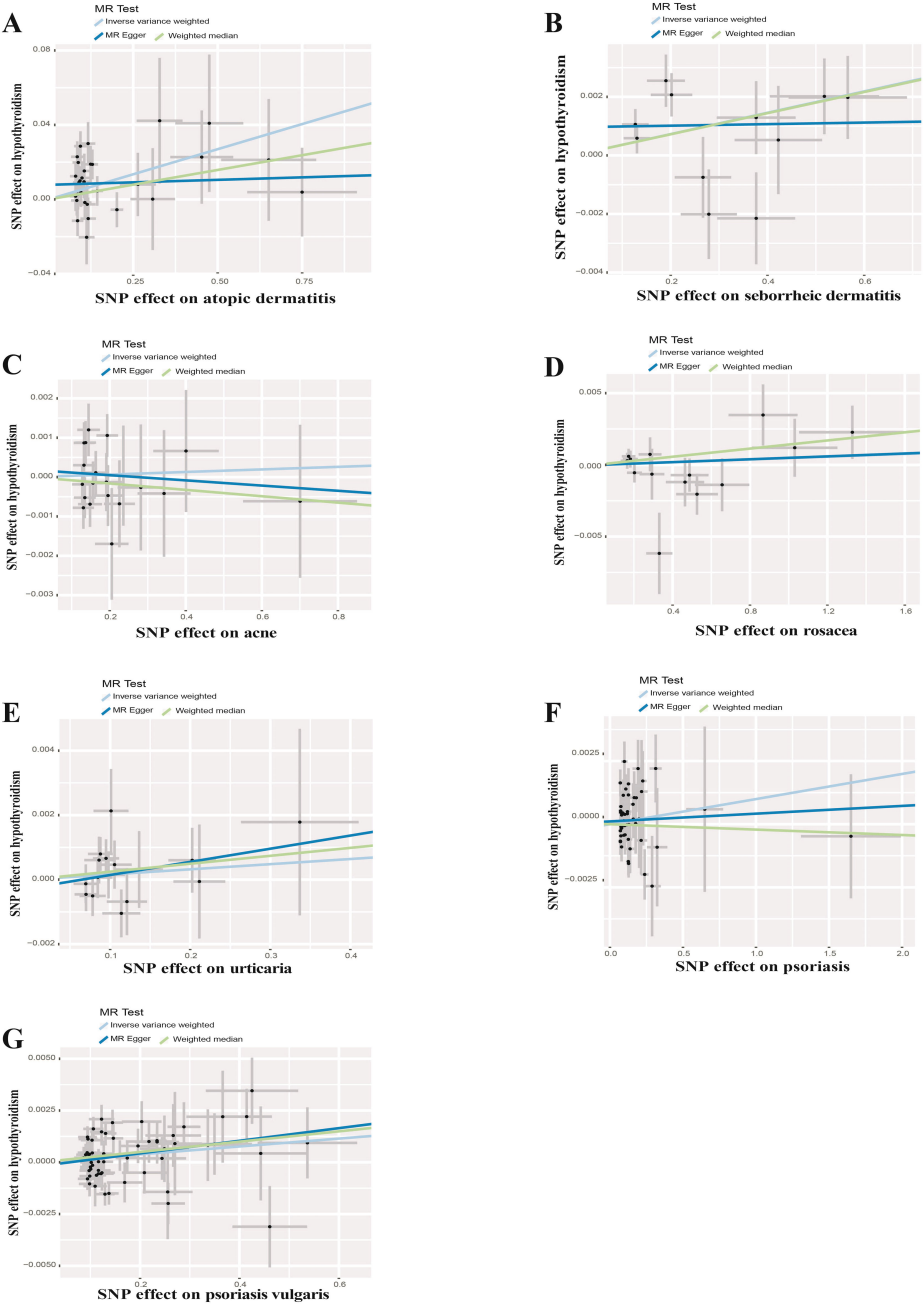
Scatter plots of causal effect estimates for inflammatory skin diseases on hypothyroidism. **(A)** Atopic dermatitis on hypothyroidism **(B)** Seborrheic dermatitis on hypothyroidism **(C)** Acne on hypothyroidism **(D)** Rosacea on hypothyroidism **(E)** Urticaria on hypothyroidism **(F)** Psoriasis on hypothyroidism **(G)** Psoriasis vulgaris on hypothyroidism.

**Figure 7:**
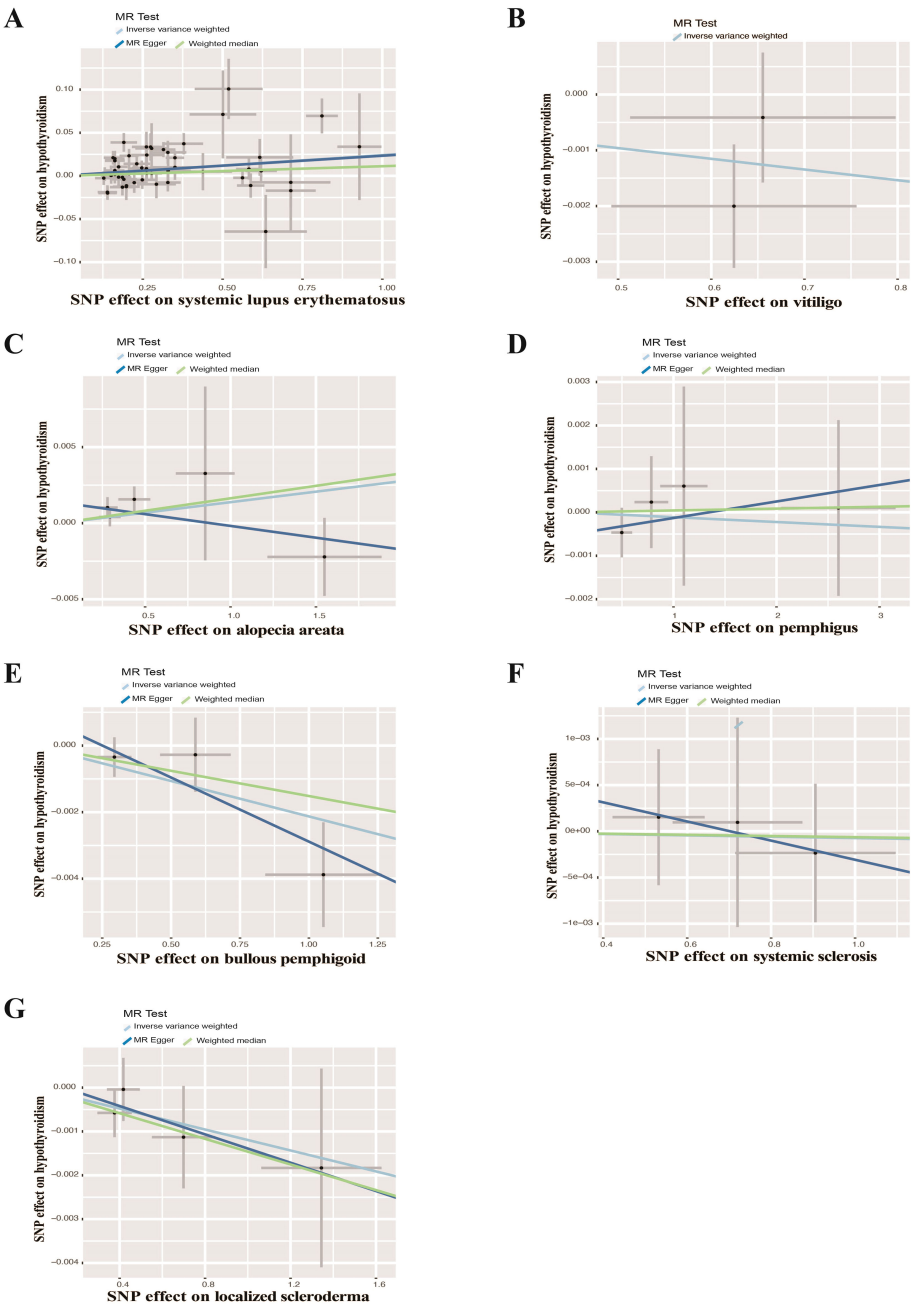
Scatter plots of causal effect estimates for autoimmune skin diseases on hypothyroidism. **(A)** Systemic lupus erythematosus on hypothyroidism **(B)** Vitiligo on hypothyroidism **(C)** Alopecia areata on hypothyroidism **(D)** Pemphigus on hypothyroidism **(E)** Bullous pemphigoid on hypothyroidism **(F)** Systemic sclerosis on hypothyroidism **(G)** Localized scleroderma on hypothyroidism.

UVMR analyses of inflammatory skin diseases showed that genetically predicted AD was associated with a 5.5% higher risk of hypothyroidism (OR = 1.055; 95%CI: 1.021-1.091; p = 0.001). The results also showed a genetic association between SD (OR = 1.004; 95%CI: 1.001-1.007; p = 0.018) and psoriasis vulgaris (OR = 1.002; 95%CI: 1.000-1.004; p = 0.024) and the risk of hypothyroidism. However, no significant genetic association was observed between acne, rosacea, urticaria, psoriasis and hypothyroidism risk. Analysis of autoimmune skin diseases showed significant positive genetic association between SLE and hypothyroidism risk, with a 2.4% higher risk (OR = 1.024; 95%CI: 1.009-1.040; p = 1.58×10^−3^). In addition, BP (OR = 0.998; 95%CI: 0.996-1.000; p = 0.036) exhibited inverse suggestive genetic association with hypothyroidism risk. No significant causal relationship was observed between genetically predisposed vitiligo, AA, pemphigus, SSc, LS, and hypothyroidism. The causal estimates were consistent across the MR Egger, weighted median and MR-PRESSO analyses (**Table 2** and **Figure 3**). Notably, none of the SNPs were removed through Steiger filtering, suggesting the correct orientation of the inferred causal relationships. Additionally, the leave-one-out analyses showed that the estimation effects between inflammatory or autoimmune skin diseases and hypothyroidism were not substantially contributed by any specific SNP (**Figures 4**, **5**). Scatter plots, with colored lines representing the slopes of different regression analyses were presented in **Figures 6**, **7**. And the symmetry of the funnel plots confirmed that there is no significant heterogeneity among SNPs, further underscoring the reliability of our findings (**Supplementary Figures S1**, **S2**).

**Table 2:** UVMR estimates for the causal associations of inflammatory or autoimmune skin diseases with hypothyroidism.

Exposure	Method	No. of SNPs or outliers^a^	OR(95%CI)	P Value^b^	R^2^(%)	F
Inflammatory skin diseases						
Atopic dermatitis	IVW-mre	30	**1.055(1.021-1.091)**	**0.001**	17.27	924.23
	Weighted median		1.032(0.992-1.074)	0.113		
	MR Egger		1.005(0.949-1.066)	0.858		
	MR-PRESSO	4	**1.055(1.021-1.091)**	**0.003**		
Seborrheic dermatitis	IVW-mre	11	**1.004(1.001-1.007)**	**0.018**	8.54	240.59
	Weighted median		**1.004(1.001-1.007)**	**0.024**		
	MR Egger		1.000(0.995-1.006)	0.929		
	MR-PRESSO	0	**1.004(1.001-1.007)**	**0.021**		
Acne	IVW-fe	20	1.000(0.999-1.002)	0.713	18.09	500.91
	Weighted median		0.999(0.997-1.002)	0.535		
	MR Egger		0.999(0.994-1.005)	0.808		
	MR-PRESSO	1	1.000(0.999-1.002)	0.723		
Rosacea	IVW-fe	14	1.000(0.999-1.002)	0.507	17.00	326.12
	Weighted median		1.001(0.999-1.003)	0.159		
	MR Egger		1.000(0.998-1.003)	0.704		
	MR-PRESSO	0	1.000(0.999-1.002)	0.554		
Urticaria	IVW-fe	16	1.002(0.998-1.005)	0.384	4.17	421.99
	Weighted median		1.002(0.998-1.007)	0.333		
	MR Egger		1.004(0.993-1.015)	0.485		
	MR-PRESSO	1	1.002(0.998-1.005)	0.569		
Psoriasis	IVW-mre	52	1.001(0.999-1.003)	0.298	19.29	1774.66
	Weighted median		1.000(0.997-1.002)	0.871		
	MR Egger		1.000(0.997-1.004)	0.856		
	MR-PRESSO	4	1.001(0.999-1.003)	0.356		
Psoriasis vulgaris	IVW-mre	60	**1.002(1.000-1.004)**	**0.024**	32.62	1880.74
	Weighted median		**1.002(1.000-1.005)**	**0.010**		
	MR Egger		1.003(0.999-1.007)	0.138		
	MR-PRESSO	1	**1.002(1.000-1.004)**	**0.031**		
Autoimmune skin diseases						
Systemic lupus erythematosus	IVW-mre	54	**1.024(1.009-1.040)**	**0.002**	6.12	3296.68
	Weighted median		1.011(0.994-1.029)	0.196		
	MR Egger		1.023(0.991-1.057)	0.168		
	MR-PRESSO	6	**1.024(1.009-1.040)**	**0.008**		
Vitiligo	IVW-fe	2	0.998(0.996-1.001)	0.126	10.75	43.68
Alopecia areata	IVW-fe	5	1.001(0.999-1.003)	0.158	11.75	117.17
	Weighted median		1.002(0.999-1.004)	0.257		
	MR Egger		0.998(0.995-1.002)	0.491		
	MR-PRESSO	0	1.001(0.999-1.004)	0.282		
Pemphigus	IVW-fe	4	1.000(0.999-1.001)	0.842	27.31	92.25
	Weighted median		1.000(0.999-1.001)	0.948		
	MR Egger		1.000(0.998-1.002)	0.735		
	MR-PRESSO	0	1.000(0.999-1.000)	0.716		
Bullous pemphigoid	IVW-fe	3	**0.998(0.996-1.000)**	**0.036**	9.42	68.46
	Weighted median		0.998(0.996-1.001)	0.247		
	MR Egger		0.996(0.992-1.001)	0.340		
Systemic sclerosis	IVW-fe	3	1.000(0.999-1.001)	0.910	31.55	67.63
	Weighted median		1.000(0.998-1.001)	0.935		
	MR Egger		0.999(0.993-1.004)	0.775		
Localized scleroderma	IVW-fe	4	0.999(0.997-1.001)	0.142	18.01	96.01
	Weighted median		0.999(0.997-1.000)	0.113		
	MR Egger		0.998(0.994-1.003)	0.527		
	MR-PRESSO	0	**0.999(0.998-0.999)**	**0.040**		

SNP, single nucleotide polymorphism; OR, odd ratio; CI, confidence interval; MR, mendelian randomization; IVW, inverse-variance weighted; mre, multiplicative-random-effects; fe, fixed-effects; PRESSO, Pleiotropy RESidual Sum and Outlier; F, F statistics; R^2^, phenotype variance explained by genetics. a. The numbers of SNPs used as IVs in the IVW, weighted median and MR Egger methods and the number of outliers identified and excluded in the MR PRESSO method. b. All data with p <0.05 are in bold.

### Causal effects of inflammatory and autoimmune skin diseases on hyperthyroidism

UVMR analysis of the effects of inflammatory or autoimmune skin diseases on hyperthyroidism showed no significant genetically predicted associations (**Supplementary Table S29** and **Figure 3**). The leave-one-out test plots, scatter plots and funnel plots of causal effect estimates for inflammatory or autoimmune skin diseases on hyperthyroidism further reflected the reliability of our results (**Supplementary Figures S3**-**S8**).

### Causal effects of inflammatory and autoimmune skin diseases on hypothyroidism and hyperthyroidism after adjusting for smoking

**Figure 8:**
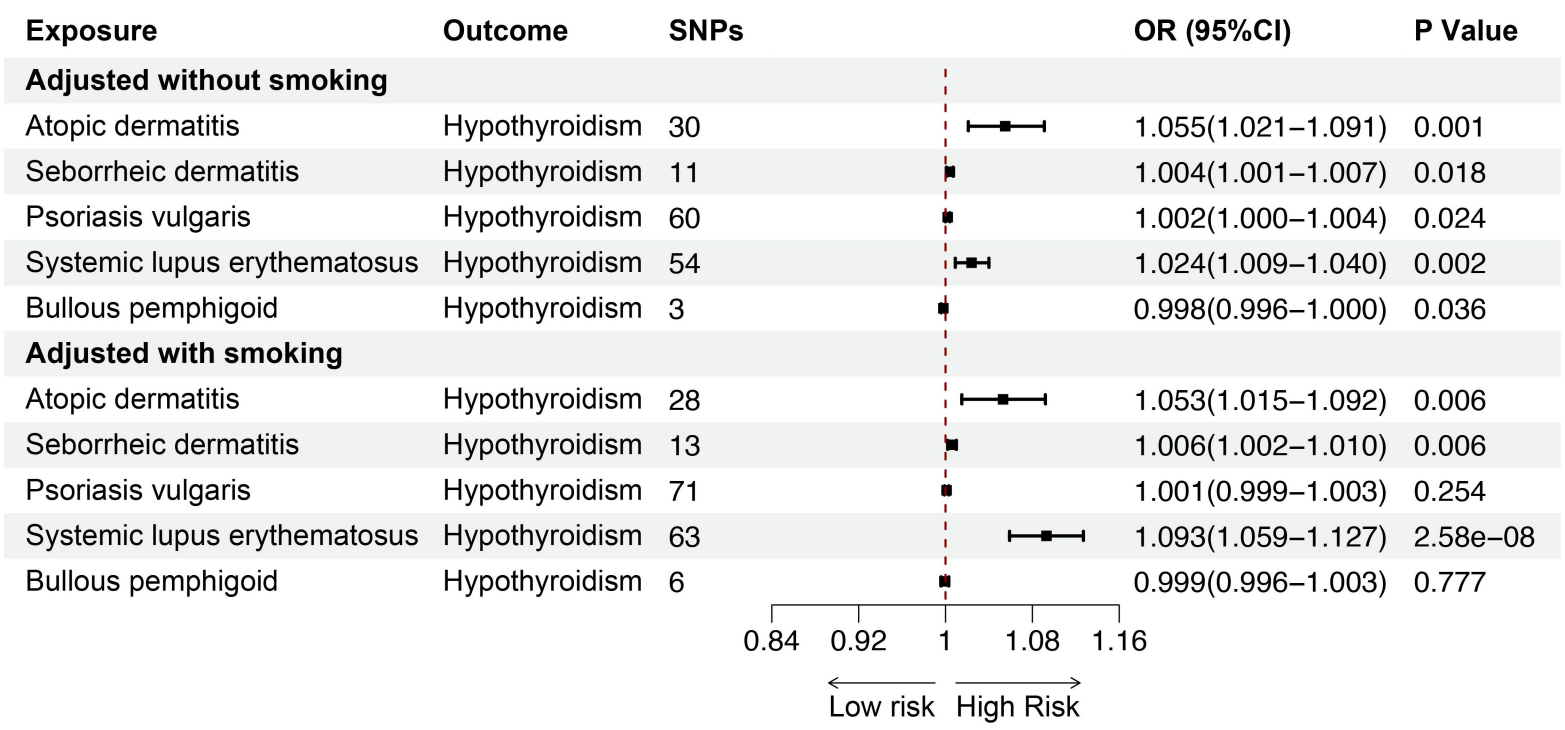
The association between inflammatory or autoimmune skin diseases and hypothyroidism adjusted without/with smoking by MVMR. MVMR, multivariate mendelian randomization; SNP, single nucleotide polymorphism; OR, odd ratio; CI, confidence interval.

**Figure 9:**
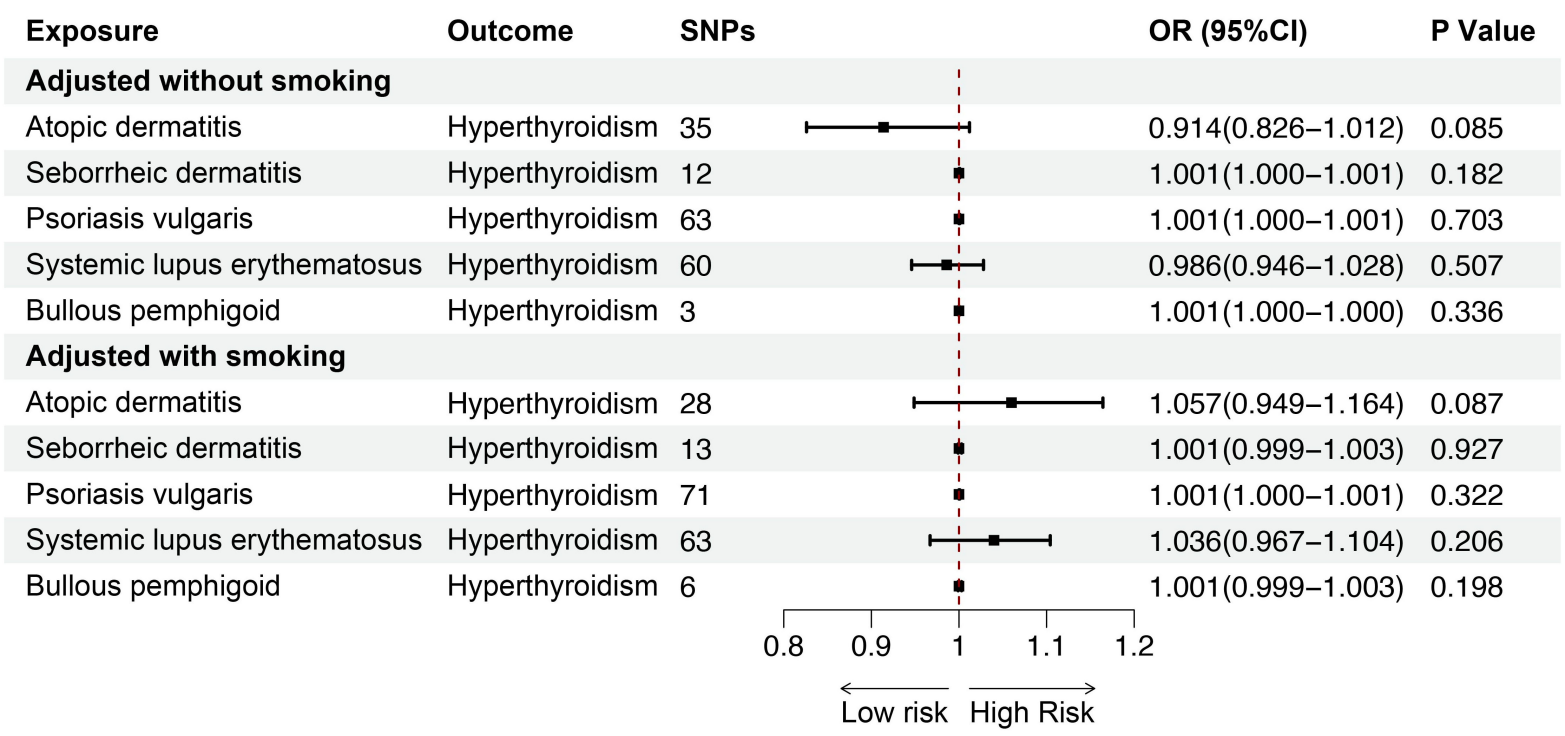
The association between inflammatory or autoimmune skin diseases and hyperthyroidism adjusted without/with smoking by MVMR. MVMR, multivariate mendelian randomization; SNP, single nucleotide polymorphism; OR, odd ratio; CI, confidence interval.

Smoking is a key risk factor for skin diseases and can affect thyroid function, so we performed MVMR for AD, SD, psoriasis vulgaris, SLE, BP after adjusting for smoking. The MVMR results showed positive associations between AD (OR = 1.053; 95%CI: 1.015-1.092; p = 0.006) and SD (OR = 1.006; 95%CI: 1.002-1.010; p = 0.006), and SLE (OR = 1.093; 95%CI: 1.059-1.127; p < 0.001) and hypothyroidism, indicating the robustness of the results. Although no significant genetic association was observed between psoriasis vulgaris, BP and hypothyroidism after adjusting for genetically predicted smoking, the findings were consistent with the direction of the estimated effect shown in the UVMR analyses (**Figure 8**). Furthermore, we conducted MVMR for the above mentioned five skin diseases and hyperthyroidism, finding no significant causal relationship between AD, SD, psoriasis vulgaris, SLE, BP and hyperthyroidism after adjusting for smoking (**Figure 9**).

### Sensitivity analysis

In all MR-Egger intercept test, there was no significant deviation from zero (p > 0.05), indicating no unbalanced horizontal pleiotropy between IVs and outcomes (**Supplementary Tables S30**-**S31**). In addition, the MR-PRESSO global test revealed that the existence of horizontal pleiotropy in some analyses, but the correlations remained consistent after eliminating outliers. However, Cochran’s Q indicated heterogeneity between inflammatory or autoimmune skin diseases-related phenotypes and hypothyroidism (**Supplementary Tables S30**-**S31**). Consequently, we adopted the multiplicative random effects IVW model to assess the associations.

## Discussion

This study offers valuable insights into the causal relationships between common inflammatory or autoimmune skin conditions and thyroid dysfunction through UVMR and MVMR analyses. The findings revealed positive causal effects of AD, SLE, SD, and psoriasis vulgaris and negative causal effects of BP on hypothyroidism risk. The positive association between AD, SLE, and SD and hypothyroidism was observed even after adjusting for smoking, indicating the robustness and reliability of the results.

Thyroid diseases elicit various dermatologic manifestations, so we mainly focused on investigating the causal effects of inflammatory or autoimmune skin diseases on thyroid conditions (10). The findings suggested that genetic predisposition to AD was linked to an elevated risk of hypothyroidism, consistent with findings from a recent National Health and Nutrition Examination Survey comprising 10,060 American participants (21). The potential association between AD and thyroid disorders may be attributed to the dysregulation of intestinal γδ T cells caused by childhood food allergies in patients with AD (22). This dysregulation of intestinal γδ T cells may lead to the suppression of regulatory T (Treg) cells through the IL-23 inflammatory cytokine pathway, resulting in development of autoimmune disorders in AD and thyroid conditions (23). Furthermore, according to the latest study by Ma EZ et al., enriched metabolites in patients with AD involved in the synthesis of thyroid hormones, which can modulate immune pathways, so AD and thyroid diseases may have common immune-metabolomics pathways (24).

This study showed that SLE was associated with a higher risk of hypothyroidism, aligning with the findings from previous observational and MR studies (25, 26). Increasing data demonstrated that the biological relationship between SLE and thyroid diseases should be established. Firstly, studies reported a higher level of thyroid autoantibodies, including thyroid peroxidase antibody (TPOAb) and thyroglobulin antibody (TGAb), in patients with SLE compared with healthy controls (27). Another study showed that SLE patients with anti-Smith antibodies were more likely to develop mild hypothyroidism (28). The above evidence confirmed that SLE and AITD, systemic and organ-specific autoimmune diseases, can be immunologically associated through autoantibodies. Secondly, the imbalance between proinflammatory mediators mediated by similar Th1 immune response in SLE and AITD may be the immunopathogenic basis of the link between these two diseases (29). Thirdly, patients with SLE have an increased susceptibility of developing AITD due to shared genetic mutations, such as the R620W polymorphism of the protein tyrosine phosphatase nonreceptor type 22 (PTPN22) gene (30).

The present results showed a suggestive association between SD and higher risk of hypothyroidism. One possible explanation of this association is the bystander activation theory, linking infections to the onset of autoimmune diseases (31). We hypothesized that after Malassezia colonization which acted as the main causative pathogen of SD, interacted with the skin surface through the release of free fatty acids and lipid peroxides, further impairing the skin barrier and activating inflammatory responses (32). This inflammatory background may lead to the activation of thyroid cells in SD patients infected with Malassezia, which resulted in secretion of pro-inflammatory cytokines and chemokines and recruitment of abundant autoreactive lymphocytes to infiltrate the thyroid gland, ultimately initiating and promoting the autoimmune process.

Two large observational cohort studies previously provided inconsistent findings on the susceptibility of patients with psoriasis to thyroid diseases. The results from one study indicated that the risk of thyroid disease was increased in patients with psoriasis (33), whereas the other study reported no significant difference in thyroid disease risk between psoriasis patients and healthy controls (34). Notably, our MR study based on genetic data revealed that psoriasis vulgaris rather than psoriasis potentially increased the risk of hypothyroidism, so the connection between different subtypes of psoriasis and the risk of thyroid dysfunction should be explored further. Scholars are currently investigating the pathogenic pathways implicated in the relationship between psoriasis and thyroid diseases, including the overlap of Th1 immune response with IL-23/Th17 axis (35), shared genetic susceptibility loci (Psoriasis susceptibility 1 candidate 1 (PSORS1C1) gene (36), and Tumor necrosis factor α-induced protein 3 (TNFAIP3) gene) (37), the miRNAome of psoriatic skin functionally enriching in thyroid hormone signaling (38), recognition of thyroid antigens by autoantibodies in patients with psoriasis (39), and vitamin D and omega-3 fatty acid deficiencies (40).

Previous controlled studies found that the association of BP with thyroid disorders is surrounded by a large inconclusiveness (41, 42). Although our results revealed that BP had a slight protective causal effect on hypothyroidism, the results need to be interpreted with caution because of few BP cases patients among the study participants and the unknown severity of BP. In addition, the presence of autoimmune comorbidities might exaggerate the causal impact of BP on the risk of thyroid dysfunction in cross-sectional studies.

Remarkably, previous studies reported that the high prevalence rates of thyroid diseases in patients with vitiligo, especially hypothyroidism (43). This phenomenon may be due to the IFN-γ/TNF-α-CXCL9/10 pathway played a central role in mediating the overlapping autoimmune imbalances in vitiligo and thyroid diseases (44). CXCR3, which is highly expressed on CD4^+^ T cells and effector CD8^+^ T cells, is the common receptor for CXCL9 and CXCL10. Therefore, pathogenic T cells were subsequently recruited and activated, secreting autoantibodies and pro-inflammatory cytokines to attack the skin and thyroid (45). However, a correlation was not observed between vitiligo and thyroid diseases in our study. This might be because the analysis could not be stratified for demographic characteristics or disease subtypes due to the lack of individual data in the GWAS database the analysis, even though vitiligo patients with positive serology or skin involvement exceeding 5% of body surface area were known to be more likely to develop thyroid disorders (46). Furthermore, only the serum levels of thyroid antibodies were abnormally elevated in many patients with vitiligo, but such GWAS databases have not yet been published for further exploration.

Our study showed no significant association of thyroid diseases with AA, which is consistent with the meta-analysis recently published by Kinoshita-Ise M et al (28). However, it is important to note that we cannot conclude based on the current results that hyperthyroidism or hypothyroidism are unrelated to AA. First of all, given that human leukocyte antigen (HLA) polymorphisms may act as main risk factors in the development of AA (47), plus HLA class II haplotypes susceptible or resistant to AA and thyroid autoimmunity (48). Therefore, the non-HLA loci shared by AA and thyroid dysfunction, also known as genetic susceptibility, may be the basis for the comorbidity of the two diseases. In addition to genetic factors, sharing epigenetic, immunological, drug, and environmental factors have been proposed as possible contributors to this association (49). Second and the most importantly, in our study, a clear distinction between overt and subclinical thyroid diseases was not made, which may obscure the relationship between AA and thyroid dysfunction. Truly, most previous controlled studies have only demonstrated that the levels of free triiodothyronine, free thyroxine, thyrotropin, TPOAb and TGAb were prone to abnormalities in patients with AA, especially severe AA (28, 50). In conclusion, although our analyses have not yet found a causal relationship between AA and thyroid diseases, the potential risk of patients with AA suffering from thyroid disorders in the future should be emphasized.

Previous studies documented an elevated risk of developing thyroid diseases in patients with urticaria, pemphigus, and SSc (51–53), but the incidence of thyroid dysfunction in patients with acne and rosacea is controversial (54–57). In the present study, the results did not show a causal relationship between acne, rosacea, urticaria, pemphigus, SSc and LS and either hyperthyroidism or hypothyroidism. Several factors may contribute to the inconsistency between the current MR findings and results from previous observational research. First, observational studies can merely illuminate the co-occurrence of inflammatory or autoimmune skin diseases and thyroid disorders, so they cannot establish the sequence or establish a causal association between the two conditions. The MR analyses in this study were based on genetic predictions and the results revealed that acne, rosacea, urticaria, SSc and LS do not predispose individuals to an elevated risk of thyroid diseases. Secondly, cross-sectional studies often comprise participants with multiple coexisting autoimmune disorders, so it is challenging to eliminate the confounding effects of other comorbid diseases on thyroid disorders. To circumvent this challenge, we identified and excluded the SNPs implicated in concurrent autoimmune diseases, such as rheumatoid arthritis, celiac disease, type 1 diabetes, and pernicious anemia to obtain “pure” causal effect estimates (15). Therefore, the results from the MR analysis indicated a significant reduction in the risk of thyroid disorders among individuals with specific inflammatory or autoimmune skin diseases, and in some instances, no significant association was identified between some skin conditions and thyroid disorders. Thirdly, recent studies revealed that glucocorticoids or immunosuppressants can modulate lymphocyte maturation, proliferation, and antibody formation by B lymphocytes, thereby affecting the prevalence of thyroid diseases (58). Inclusion of study subjects with a history of corticosteroid or immunosuppressant use may result in discordant results between MR studies and observational investigations.

It is worth noting that our results showed that both inflammatory and autoimmune skin diseases are associated with hypothyroidism but not hyperthyroidism. Differences in genetic factors, epigenetic mechanisms, and autoimmune responses between hyperthyroidism and hypothyroidism may partially explain the variation in the causal effect of these factors on hypothyroidism and hyperthyroidism (59). More importantly, hyperthyroidism exhibits apparent symptoms such as irritability, tremors, and weight loss (8), whereas hypothyroidism has subtle manifestations including fatigue, excessive sleep, and depression (7), which may be easily ignored. Therefore, clinicians should recommend that patients with inflammatory or autoimmune skin diseases undergo early and regular thyroid function screening to prevent and treat thyroid conditions that has not yet occurred or already exists.

The present study has several strengths. First, our study stands as the inaugural MR analysis to delve the causal relationship between inflammatory or autoimmune skin diseases and thyroid dysfunction using both UVMR and MVMR methods. Second, we included relatively comprehensive phenotypes of common inflammatory or autoimmune skin diseases in the MR setting. Moreover, the utilization of disparate data sources for exposure and outcome datasets mitigated the likelihood of sample overlap (60). We also performed multiple sensitivity analyses and outlier assessments to minimize the impact of pleiotropy and outliers, increasing the robustness and reliability of the findings. Third, the participants included in the GWAS studies were of European ancestry, which helped reduce population stratification bias.

The MR analyses have some limitations. First, pleiotropy in the MR setting was a major challenge. Thus, we employed the MR-Egger intercept evaluation and MR-PRESSO to detect and exclude the horizontal pleiotropy. The two tests showed limited indications of pleiotropy, indicating that the results were reliable. Second, significant heterogeneity was observed in some of the MR analyses, but our main results presented homogeneity in the direction and magnitude of effect estimates when weighted median, MR-Egger, multiplicative-random-effects IVW, and MR-PRESSO analyses were conducted after excluding any potential outlier SNPs. Third, our results drew upon GWAS data from mainly individuals of European descent. Therefore, the conclusions obtained in this study should be carefully generalized to other races and populations.

## Conclusion

The current study provides evidence that AD, SLE, and SD are causally associated with an increased risk of hypothyroidism. The pathophysiologic mechanisms underlying the crosstalk between skin diseases and thyroid dysfunction should be explored in further investigations. The findings from this research provide vital information for clinicians that measures and concerted efforts for follow-up of thyroid function and early intervention of thyroid diseases should be considered in subjects diagnosed with AD, SLE, and SD, particularly patients with other autoimmune comorbidities.

## Data Availability

The original contributions presented in the study are included in the article/**Supplementary Material**. Further inquiries can be directed to the corresponding authors.
