# Supplementary material for: The causal effects of inflammatory and autoimmune skin diseases on thyroid diseases: evidence from Mendelian randomization study
Source: Front Endocrinol (Lausanne). 2024 Sep 2;15:1388047. doi: 10.3389/fendo.2024.1388047 (PMC11402664; doi:10.3389/fendo.2024.1388047)
Supplement: Supplementary file 2 [file DataSheet2.docx]

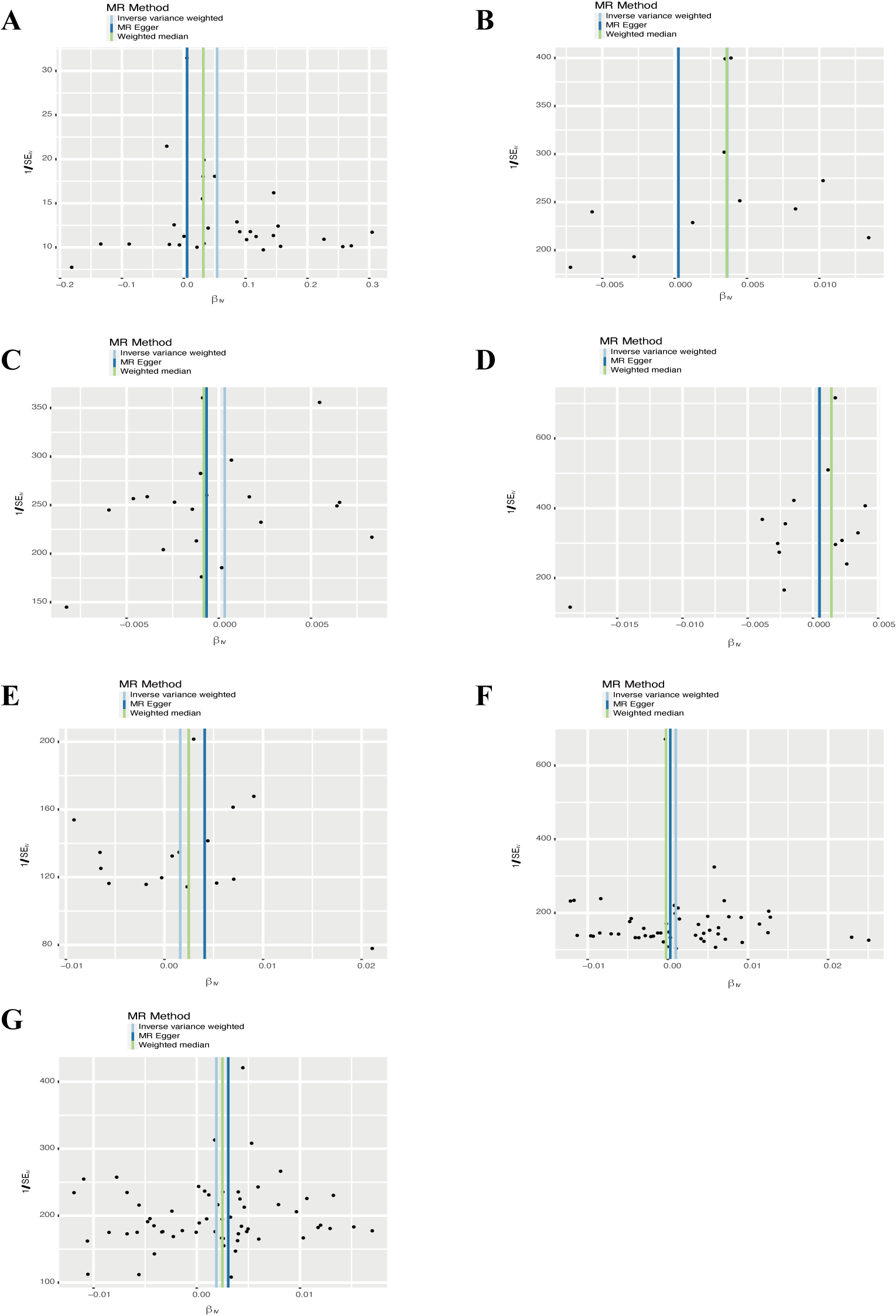


**Supplemental Figure 1** Funnel plots of causal effect estimates for inflammatory skin diseases on hypothyroidism. (A) Atopic dermatitis on hypothyroidism (B) Seborrheic dermatitis on hypothyroidism (C) Acne on hypothyroidism (D) Rosacea on hypothyroidism (E) Urticaria on hypothyroidism (F) Psoriasis on hypothyroidism (G) Psoriasis vulgaris on hypothyroidism


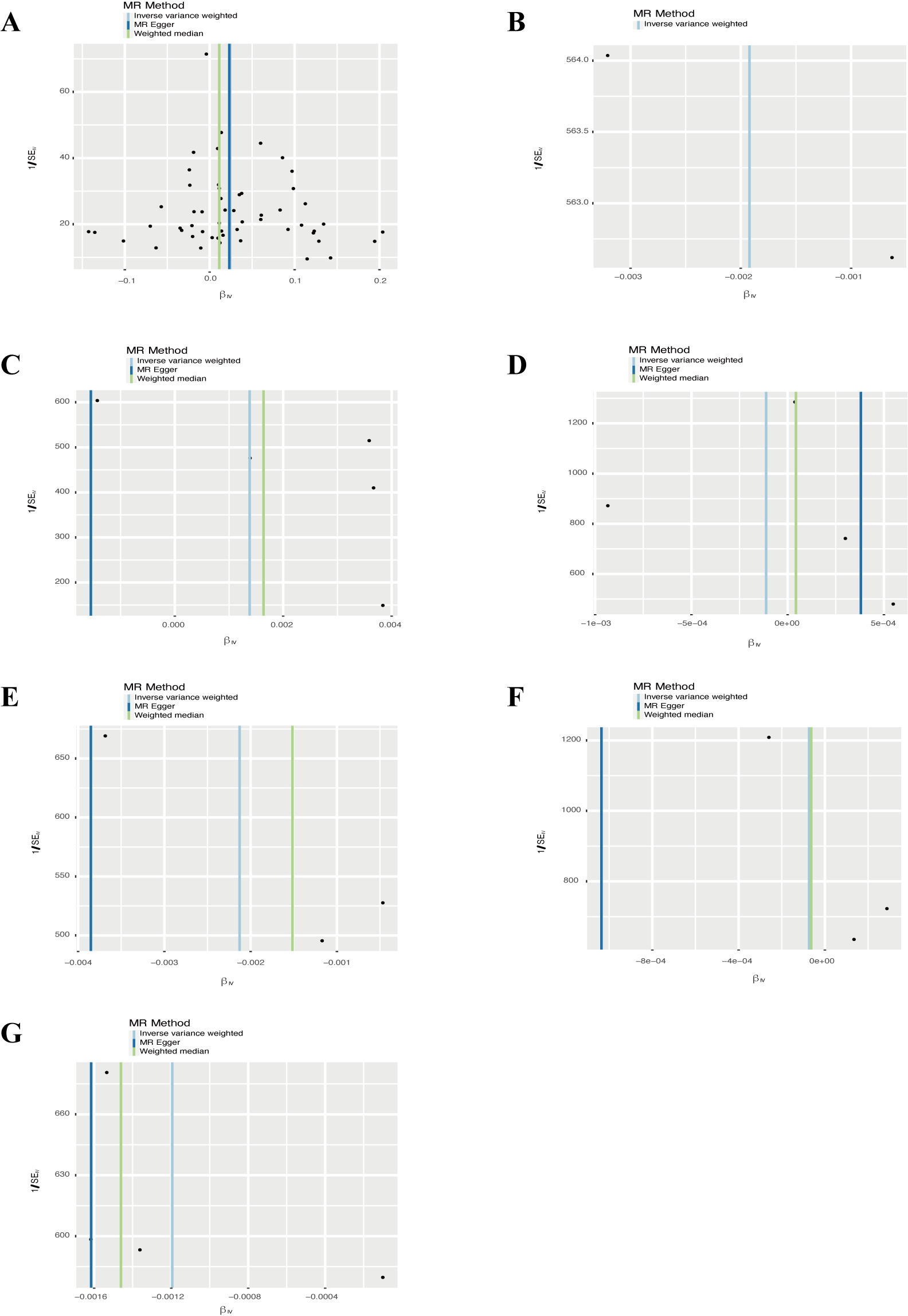


**Supplemental Figure 2** Funnel plots of causal effect estimates for autoimmune skin diseases on hypothyroidism. (A) Systemic lupus erythematosus on hypothyroidism (B) Vitiligo on hypothyroidism (C) Alopecia areata on hypothyroidism (D) Pemphigus on hypothyroidism (E) Bullous pemphigoid on hypothyroidism (F) Systemic sclerosis on hypothyroidism (G) Localized scleroderma on hypothyroidism


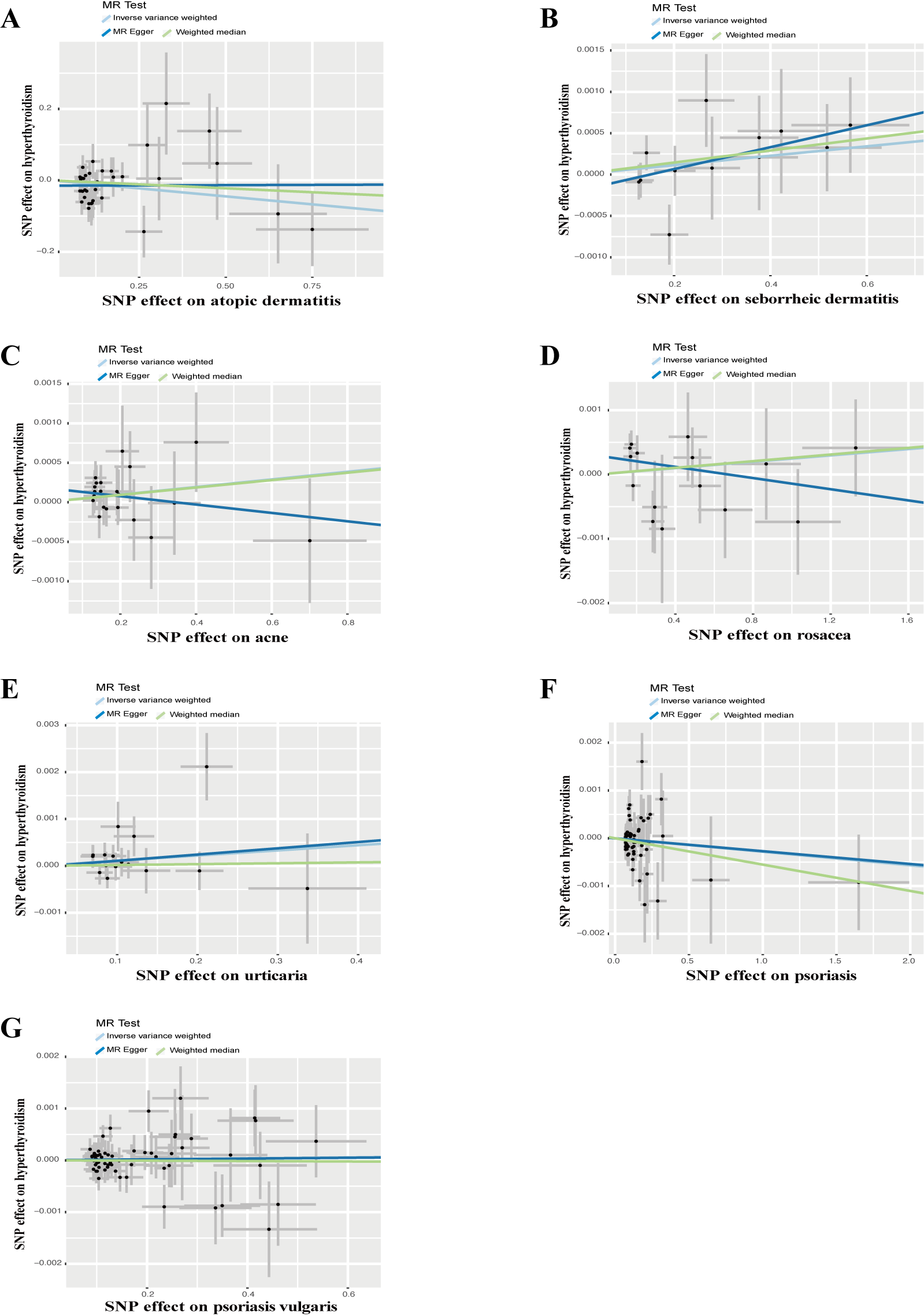


**Supplemental Figure 3** Scatter plots of causal effect estimates for inflammatory skin diseases on hyperthyroidism. (A) Atopic dermatitis on hyperthyroidism (B) Seborrheic dermatitis on hyperthyroidism (C) Acne on hyperthyroidism (D) Rosacea on hyperthyroidism (E) Urticaria on hyperthyroidism (F) Psoriasis on hyperthyroidism (G) Psoriasis vulgaris on hyperthyroidism


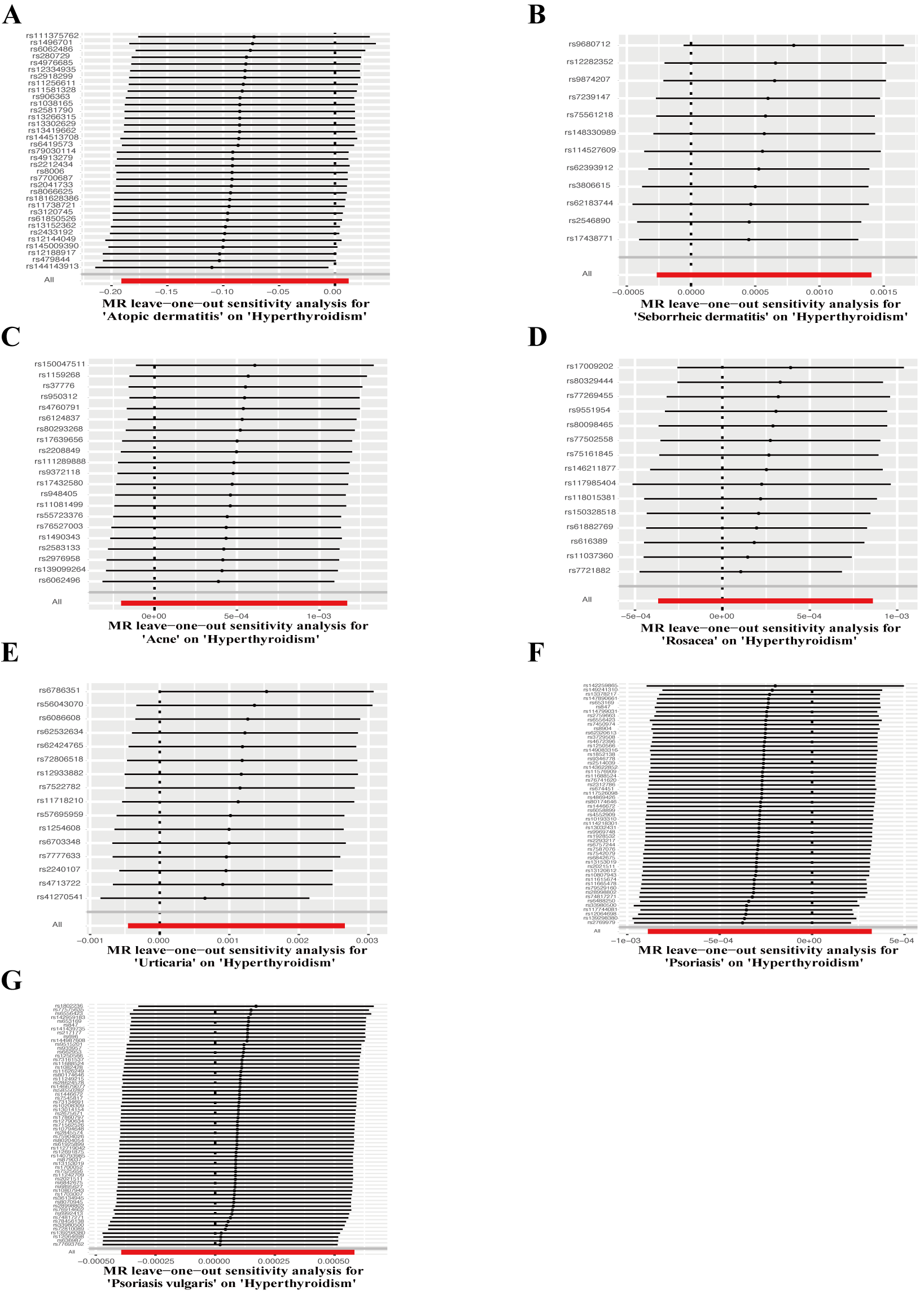


**Supplemental Figure 4** Leave-one-out test plots of causal effect estimates for inflammatory skin diseases on hyperthyroidism. (A) Atopic dermatitis on hyperthyroidism (B) Seborrheic dermatitis on hyperthyroidism (C) Acne on hyperthyroidism (D) Rosacea on hyperthyroidism (E) Urticaria on hyperthyroidism (F) Psoriasis on hyperthyroidism (G) Psoriasis vulgaris on hyperthyroidism


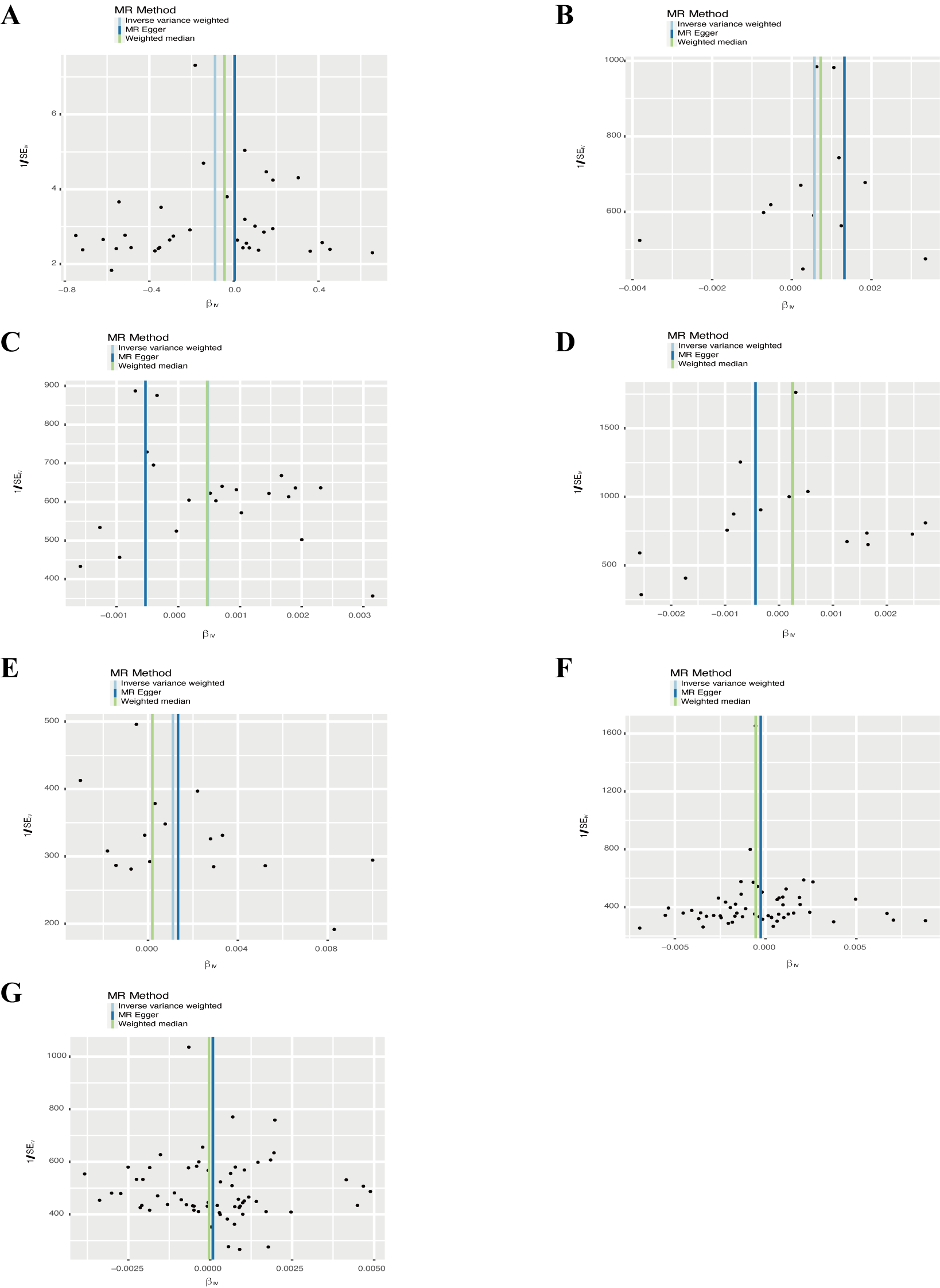


**Supplemental Figure 5** Funnel plots of causal effect estimates for inflammatory skin diseases on hyperthyroidism. (A) Atopic dermatitis on hyperthyroidism (B) Seborrheic dermatitis on hyperthyroidism (C) Acne on hyperthyroidism (D) Rosacea on hyperthyroidism (E) Urticaria on hyperthyroidism (F) Psoriasis on hyperthyroidism (G) Psoriasis vulgaris on hyperthyroidism

**
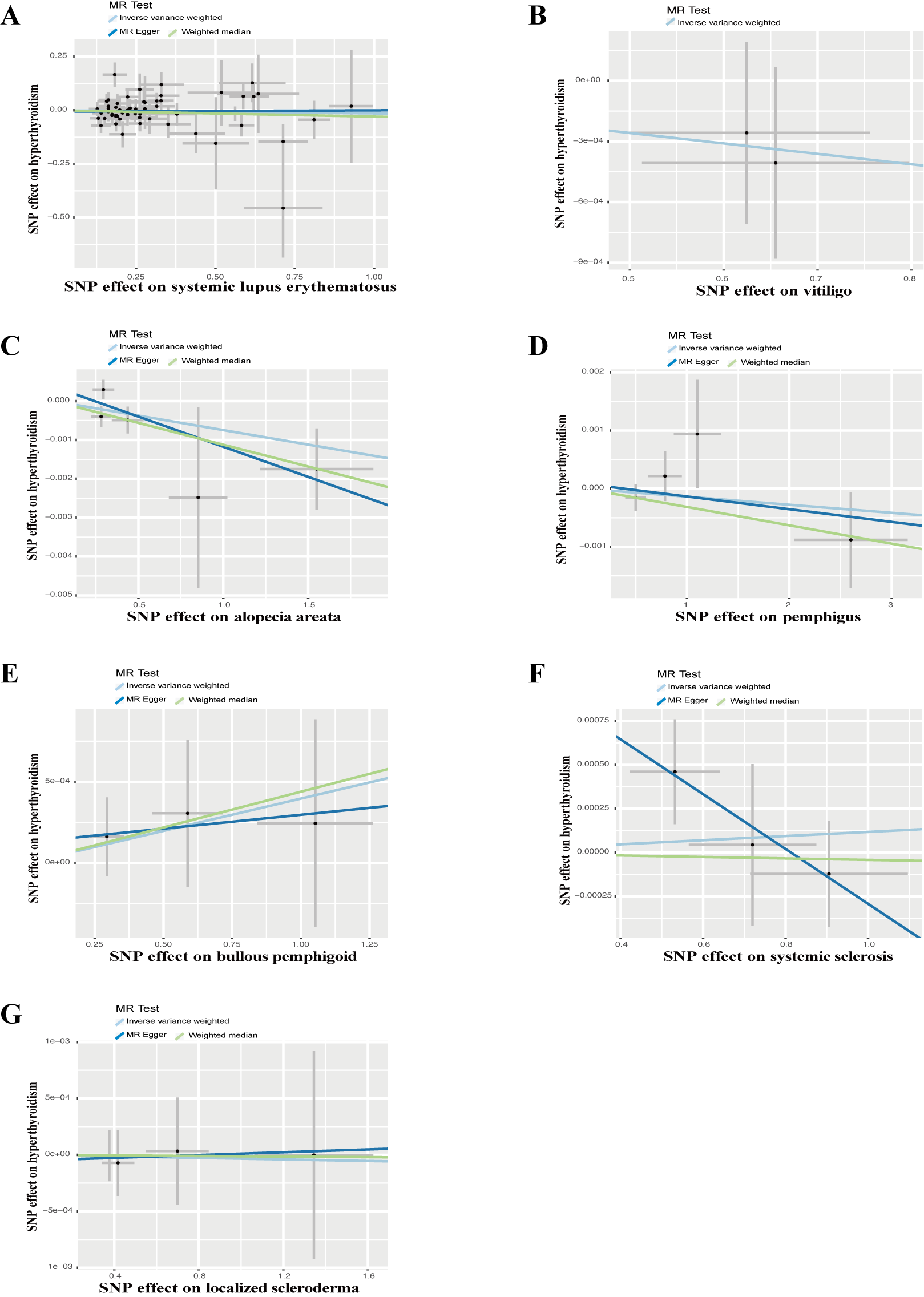
**

**Supplemental Figure 6** Scatter plots of causal effect estimates for autoimmune skin diseases on hyperthyroidism. (A) Systemic lupus erythematosus on hyperthyroidism (B) Vitiligo on hyperthyroidism (C) Alopecia areata on hyperthyroidism (D) Pemphigus on hyperthyroidism (E) Bullous pemphigoid on hyperthyroidism (F) Systemic sclerosis on hyperthyroidism (G) Localized scleroderma on hyperthyroidism

**
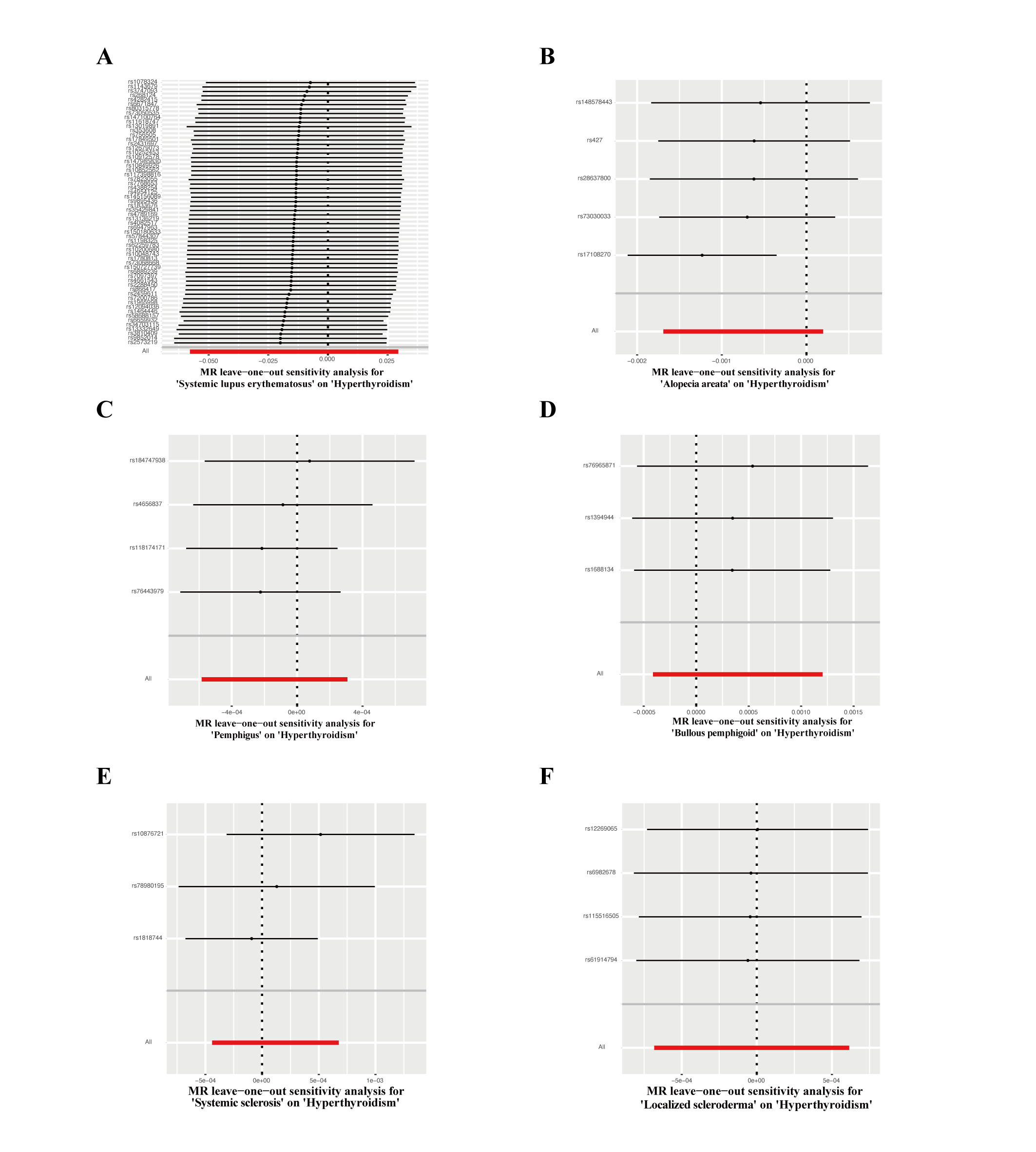
**

**Supplemental Figure 7** Leave-one-out test plots of causal effect estimates for autoimmune skin diseases on hyperthyroidism. (A) Systemic lupus erythematosus on hyperthyroidism (B) Alopecia areata on hyperthyroidism (C) Pemphigus on hyperthyroidism (D) Bullous pemphigoid on hyperthyroidism (E) Systemic sclerosis on hyperthyroidism (F) Localized scleroderma on hyperthyroidism

**
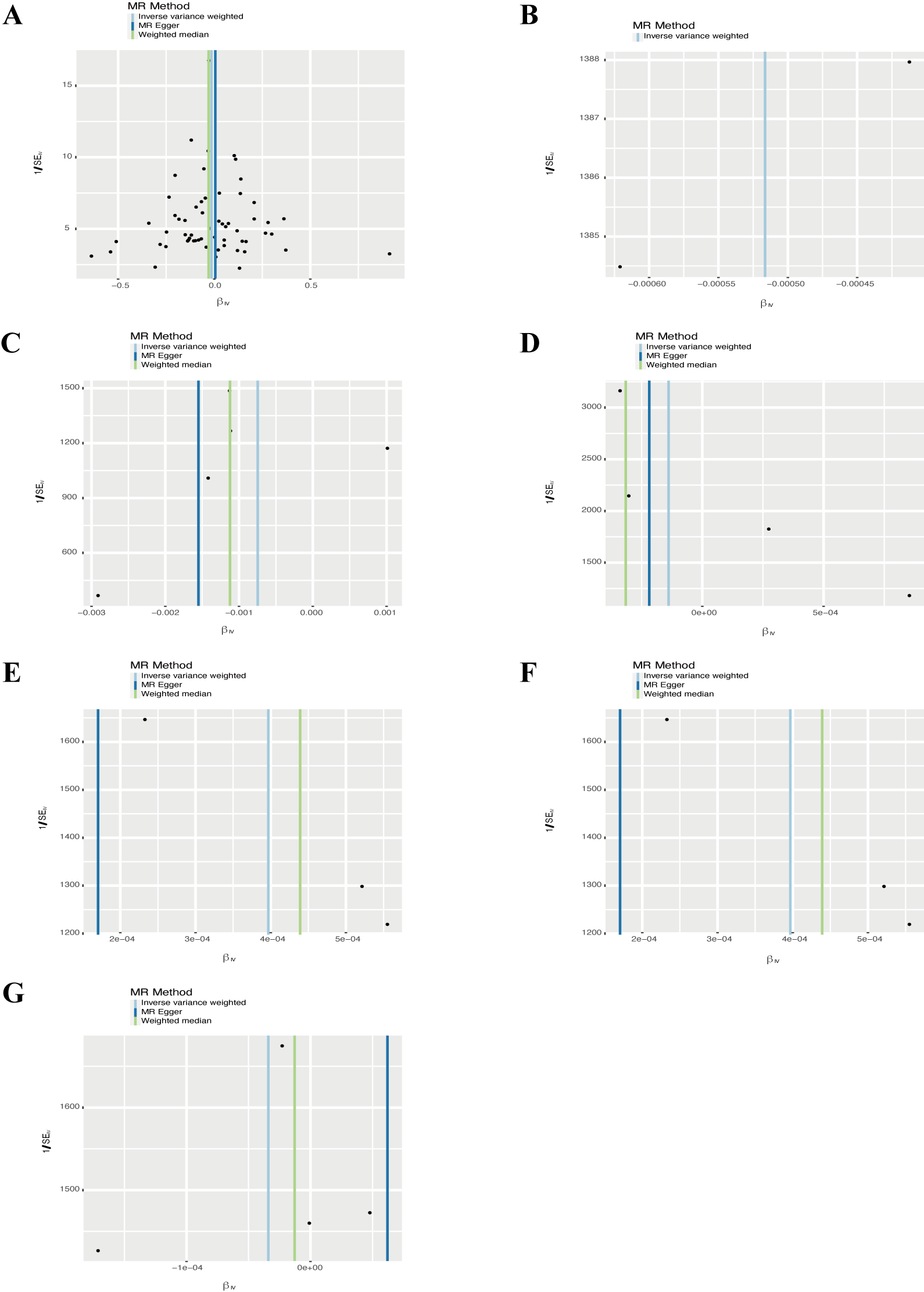
**

**Supplemental Figure 8** Funnel plots of causal effect estimates for autoimmune skin diseases on hyperthyroidism. (A) Systemic lupus erythematosus on hyperthyroidism (B) Vitiligo on hyperthyroidism (C) Alopecia areata on hyperthyroidism (D) Pemphigus on hyperthyroidism (E) Bullous pemphigoid on hyperthyroidism (F) Systemic sclerosis on hyperthyroidism (G) Localized scleroderma on hyperthyroidism
